# Real-time open-vocabulary perception for mobile robots on edge devices: a systematic analysis of the accuracy-latency trade-off

**DOI:** 10.3389/frobt.2025.1693988

**Published:** 2025-10-21

**Authors:** Jongyoon Park, Pileun Kim, Daeil Ko

**Affiliations:** 1 Intelligence Robotics and Autonomous Mobility, Department of Artificial Intelligent, Korea Aerospace University, Goyang-si, Republic of Korea; 2 Intelligence Robotics and Autonomous Mobility, Department of Autonomous Vehicle Engineering, Korea Aerospace University, Goyang-si, Republic of Korea

**Keywords:** edge Device, zero-Shot, real-time, optimization, human-robot interaction

## Abstract

The integration of Vision-Language Models (VLMs) into autonomous systems is of growing importance for improving Human-Robot Interaction (HRI), enabling robots to operate within complex and unstructured environments and collaborate with non-expert users. For mobile robots to be effectively deployed in dynamic settings such as domestic or industrial areas, the ability to interpret and execute natural language commands is crucial. However, while VLMs offer powerful zero-shot, open-vocabulary recognition capabilities, their high computational cost presents a significant challenge for real-time performance on resource-constrained edge devices. This study provides a systematic analysis of the trade-offs involved in optimizing a real-time robotic perception pipeline on the NVIDIA Jetson AGX Orin 64GB platform. We investigate the relationship between accuracy and latency by evaluating combinations of two open-vocabulary detection models and two prompt-based segmentation models. Each pipeline is optimized using various precision levels (FP32, FP16, and Best) via NVIDIA TensorRT. We present a quantitative comparison of the mean Intersection over Union (mIoU) and latency for each configuration, offering practical insights and benchmarks for researchers and developers deploying these advanced models on embedded systems.

## 1 Introduction

As the paradigm of robotics expands beyond highly controlled industrial environments and into the daily lives of humans, a central challenge has emerged: enabling non-expert users to intuitively communicate and collaborate with robots. The realization of this vision hinges on surmounting the fundamental communication barrier between humans and machines, which can be achieved when robots move beyond complex programming or formalized command structures to directly understand and execute natural language. Therefore, the ability for a robot to accurately connect, or “ground,” linguistic concepts to the physical, visual world is an essential prerequisite.

Vision-Language Models (VLMs), pre-trained on large-scale datasets, have arisen as an effective solution to this language-vision grounding problem (Radford et al., 2021). VLMs provide open-vocabulary recognition, which allows them to identify objects described by arbitrary text in a zero-shot manner, extending far beyond a limited set of predefined categories. This transformative capability grants robots the potential to interact with novel objects in unpredictable environments, positioning them as a key enabling technology for truly flexible and adaptive Human-Robot Interaction (HRI).

However, the powerful performance of VLMs is accompanied by immense computational costs, creating a direct conflict with the inherent resource constraints of edge devices embedded in real-world robotic systems. Even state-of-the-art edge platforms, such as the NVIDIA Jetson AGX Orin, have limitations in compute and memory bandwidth that cannot match the demands of server-grade GPUs required by most VLMs. This results in a “real-time perception bottleneck,” where a robot’s intelligence can leverage state-of-the-art AI, but its real-time responsiveness is degraded by hardware limitations. Securing a response rate of over 10 FPS, the minimum requirement for seamless human interaction, presents a significant optimization challenge when deploying VLMs in edge environments.

This study presents a systematic approach to bridge this critical “real-time gap.” To this end, we conduct a comprehensive benchmarking study of four distinct open-vocabulary instance segmentation pipelines on the NVIDIA Jetson AGX Orin platform. This focus on an efficient perception module serves as a critical foundational step for more complex, downstream robotics tasks, such as those envisioned in Vision-Language-Action (VLA) frameworks (Brohan et al., 2023; Lee et al., 2024). Our contributions are as follows:

• Systematic Benchmarking of Diverse Pipeline Philosophies: We provide a detailed comparative analysis of pipelines constructed from two different detector philosophies a VLM based approach (NanoOWL) and a highly efficient YOLO-based model (YOLO-World) paired with two segmentation model strategies: knowledge distillation (NanoSAM) and efficient architecture design (EfficientViT-SAM).
• In-depth Analysis of Edge Device Optimization: Beyond surface-level performance metrics, we quantitatively investigate the practical trade-offs between segmentation accuracy (mIoU) and end-to-end latency through NVIDIA TensorRT optimization across various precision configurations (FP32, FP16 and Best).
• Actionable Framework for Real-World Robotics: Based on our empirical results, we provide the robotics community with a clear and actionable framework for selecting the optimal perception pipeline and precision strategy according to the specific requirements of HRI scenarios (e.g., prioritizing response speed *versus* recognition accuracy).

Ultimately, the objective of this research is not merely to accelerate a single model, but to explore the multi-dimensional design space of model architectures, optimization techniques, and precision levels to find practical solutions that enable fluid, real-time interaction between humans and robots on resource-constrained platforms.

## 2 Related work

### 2.1 Open-vocabulary object detection

Open-vocabulary object detection aims to detect and classify objects corresponding to arbitrary natural language text queries, moving beyond a predefined and limited set of classes (Gu et al., 2022). This capability is a fundamental prerequisite for robots to understand and interact with diverse user commands in unstructured environments. This paper surveys two representative approaches with distinct design philosophies: one centered on the generality achieved by adapting large-scale Vision-Language Models, and the other on the efficiency inherent to highly optimized detector architectures (Liu et al., 2024).

#### 2.1.1 OWL-ViT: adapting vision-language models for localization

Open-World Localization Vision Transformer (OWL-ViT) (Minderer et al., 2022) is a pioneering model that successfully adapts the powerful language-vision representations of a pre-trained CLIP model for the task of object detection. The core architectural modification in OWL-ViT involves removing the final [CLASS] token pooling from the ViT image encoder to preserve the spatial feature map. Lightweight classification and bounding box prediction heads are then attached to each grid token. The model’s zero-shot, open-vocabulary capability is realized by dynamically populating the classifier weights with text prompt embeddings generated by the CLIP text encoder. While this architecture offers excellent generalization, it has inherent limitations for real-time performance on edge devices due to the requisite interaction between image and text embeddings at inference time.

#### 2.1.2 NanoOWL: a post-hoc optimization framework for the edge

NanoOWL is not a new model architecture but rather an optimization framework designed to deploy powerful yet computationally heavy models like OWL-ViT onto resource-constrained edge devices such as the NVIDIA Jetson. It encompasses the process of converting the PyTorch-based OWL-ViT model into a highly efficient inference engine using NVIDIA TensorRT. In essence, NanoOWL represents a pragmatic approach that seeks to achieve real-time performance through *post hoc* optimization for specific hardware, while retaining the high generality of the original model.

#### 2.1.3 YOLO-world: open-vocabulary detection through efficiency-by-design

In contrast, YOLO-World represents a paradigm shift by integrating open-vocabulary capabilities into the highly efficient YOLOv8 CNN framework (Yaseen, 2024). Its key technology, the Re-parameterizable Vision-Language Path Aggregation Network (RepVL-PAN), effectively fuses visual and linguistic features (Cheng et al., 2024). Notably, YOLO-World maximizes real-time efficiency through a novel “prompt-then-detect” paradigm. This method involves pre-encoding a set of user-defined text prompts into an “offline vocabulary,” which is then integrated directly into the network weights. This eliminates the need for a text encoder during inference, drastically reducing latency. This provides a significant speed advantage compared to models like OWL-ViT that require online text encoding for every inference, and can be characterized as an “efficiency-by-design” strategy inherently well-suited for edge-device applications.

### 2.2 Prompt-based image segmentation

Prompt-based segmentation is the task of generating a precise mask for a specific object within an image, conditioned on various user inputs such as points, boxes, or text. This is essential for robotic systems to ascertain the exact geometry of a detected object for fine-grained manipulation tasks.

#### 2.2.1 Segment anything model (SAM): a foundation model for segmentation

The Segment Anything Model (SAM) is a transformative foundation model for image segmentation (Kirillov et al., 2023). Comprising a powerful ViT-H image encoder, a prompt encoder, and a mask decoder, SAM has demonstrated remarkable zero-shot generalization performance in producing high-quality segmentation masks for a wide array of prompts. However, the immense computational requirement of its ViT-H encoder makes it infeasible for deployment in real-time edge computing environments.

#### 2.2.2 NanoSAM: real-time segmentation via knowledge distillation

NanoSAM aims to achieve real-time performance for SAM on Jetson devices. It employs knowledge distillation, a training technique, to enable a lightweight ResNet-18 image encoder to mimic the outputs of a larger “teacher” model the ViT from MobileSAM (Zhang et al., 2023). Through this process, the simpler “student” CNN architecture learns to approximate the rich feature representations of the more complex teacher, achieving an effective trade-off between inference speed and segmentation accuracy.

#### 2.2.3 EfficientViT-SAM: high-performance segmentation through architectural innovation

EfficientViT-SAM offers an alternative strategy by replacing SAM’s computationally heavy ViT encoder with EfficientViT (Cai et al., 2023), a fundamentally more efficient Transformer architecture designed for hardware-aware execution. EfficientViT achieves its efficiency by leveraging techniques such as multi-scale linear attention, which reduces the complexity of the attention mechanism from quadratic $\left(O\left(N^{2}\right)\right)$ to linear $O\left(N\right)$ with respect to the input size. The training process consists of two stages: first, knowledge from the original SAM-ViT-H image encoder is distilled into the EfficientViT encoder, after which the entire model is fine-tuned end-to-end on the large-scale SA-1B dataset. This work was reported to achieve significant speed-ups with negligible performance degradation compared to the original SAM-ViT-H, and this study aims to empirically validate this claim on the NVIDIA Jetson platform.

### 2.3 Model optimization with NVIDIA TensorRT

NVIDIA TensorRT is a high-performance optimizer and runtime library for deep learning inference. It takes a trained model and generates an optimized inference engine for NVIDIA GPU architectures through various techniques, including graph optimization, layer fusion, and kernel auto-tuning.

A key feature of TensorRT, and a central optimization strategy in this study, is low-precision inference. While deep learning models are typically trained in 32-bit floating-point (FP32) precision, TensorRT can convert them to lower precisions. For NVIDIA’s Ampere and subsequent architectures, TensorRT often utilizes FP32 to accelerate operations that are nominally FP32, offering a speedup with no code changes. For further performance gains, it can convert models to 16-bit floating-point (FP16) or automatically determined best precision. This reduction in precision decreases memory usage and bandwidth requirements and leverages specialized hardware units like Tensor Cores to maximize inference throughput. However, this process involves a trade-off, as it carries the risk of some accuracy degradation. Finding the optimal precision strategy for each model and task is therefore critical to achieving both real-time performance and high accuracy on edge devices.

## 3 Methods

The experiments in this study were systematically designed to ensure reproducible and rigorous evaluation in realistic robotics application scenarios. This chapter details the architecture of the integrated perception pipeline, the model variants used for evaluation, the optimization protocol tailored for the NVIDIA Jetson AGX Orin platform, and the evaluation metrics established to quantify performance.

### 3.1 Integrated perception pipeline framework

**FIGURE 1:**
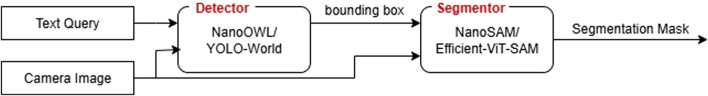
The architecture of the two-stage ‘detect-then-segment’ open-vocabulary perception pipeline. A user-provided text query and a camera image are fed into the Detector module (NanoOWL or YOLO-World) to generate a bounding box. This bounding box, along with the original image, is then passed to the Segmentor module (NanoSAM or EfficientViT-SAM) to produce the final segmentation mask.

The overall data flow of our integrated perception pipeline, which is based on a common two-stage ‘detect-then-segment’ architecture, is illustrated in Figure 1. This modular structure was adopted for its proven efficacy in open-vocabulary instance segmentation. The pipeline’s data flow is defined as follows:

• Input: A user provides the system with a natural language text prompt, P (e.g., “a red mug on the table”), and an RGB image I .
• Detection Stage: An open-vocabulary object detector D , takes the image I and prompt P as input to generate a set of bounding box proposals $B=\left\{b_{1},b_{2},\ldots ,b_{n}\right\}$ corresponding to potential instances of the described object.
• Segmentation Stage: A prompt-based image segmentation model S , uses the original image I and the bounding box $b_{i}\in B$ with the highest confidence from the detection stage as a visual prompt. It then outputs a high-quality binary segmentation mask M that precisely isolates the target object.

This perception module is designed as the core visual input for a mobile manipulator, where the final output mask M is used to determine the precise shape and location of a target object in HRI scenarios. Therefore, the real-time performance target of over 10 FPS is not an arbitrary benchmark but a crucial system requirement for fluid and natural interaction between users and robots.

### 3.2 Models and variants for evaluation

For a comprehensive analysis, this study selected a spectrum of model variants representing different sizes, theoretical performance, and underlying architectural philosophies. First, for open-vocabulary object detection, two families of models were evaluated. Representing the approach of adapting large-scale VLMs for the edge, three primary variants of NanoOWL were selected to analyze the impact of backbone scale on performance and latency: owlvit-base-patch32, owlvit-base-patch16, and owlvit-large-patch14. Representing the ‘efficiency-by-design’ philosophy, three officially provided variants of YOLO-World were evaluated to analyze the trade-off between size and zero-shot capability: YOLO-World-S and YOLO-World-X.

Next, for prompt-based image segmentation, models from two distinct strategies were chosen. Representing the model lightening strategy through knowledge distillation, NanoSAM is composed of a lightweight ResNet-18 image encoder and a MobileSAM mask decoder. In contrast, representing the optimization strategy through efficient architectural design, five variants of EfficientViT-SAM were included to evaluate performance at various operating points: L0, L1, L2, XL0, and XL1.

### 3.3 Edge device optimization protocol

All experiments were conducted on an NVIDIA Jetson AGX Orin 64GB Developer Kit set to maximum performance mode (MAXN) to preclude thermal throttling as a potential performance variable. To ensure consistency and reproducibility, the software stack was standardized on JetPack 6.0, which includes CUDA 12.2 and TensorRT 8.6.2.

For each model variant, a suite of optimized TensorRT engines (.engine files) was generated via an ONNX intermediate representation using the trtexec command-line tool. The following precision configurations were systematically evaluated. FP32 was used as the baseline precision, offering the highest accuracy with acceleration on Tensor Cores. FP16 was evaluated for its potential to yield significant speedups with minimal accuracy loss by leveraging the Orin GPU’s Tensor Cores. Finally, the best mode was used as a mixed-precision strategy, where TensorRT profiles all available precision implementations for each layer and heuristically selects the fastest one. This is a dynamic optimization process that aims for the lowest achievable latency, potentially resulting in a heterogeneous precision configuration across layers.

### 3.4 Evaluation protocol and metrics

The choice of dataset is critical for evaluating the language understanding capabilities of open-vocabulary models. This study utilizes the RefCOCO + dataset (Yu et al., 2016), a benchmark for referring expression segmentation. Unlike category-based datasets, RefCOCO + provides complex linguistic phrases that uniquely identify an object (e.g., “the man in the red shirt”) and its corresponding ground-truth mask. It is therefore an ideal benchmark for directly assessing a model’s ability to “ground” nuanced language to visual features.

The primary performance metrics are accuracy and latency. Accuracy is measured using the mIoU (mean Intersection over Union), calculated between the generated segmentation mask and the ground-truth mask from the RefCOCO + dataset to evaluate pixel-level precision. Latency is quantified as the end-to-end pipeline latency in milliseconds, measuring the wall-clock time from receiving the image and text prompt to outputting the final mask, inclusive of all preprocessing, model inference, and postprocessing stages. Measurements are averaged over the validation set after an initial 20 warm-up inferences, with throughput reported in Frames Per Second (FPS).

Furthermore, component-level latencies for the detection and segmentation stages are reported separately to identify performance bottlenecks.

## 4 Results

This section presents the empirical results of the benchmarking study. The analysis begins with the performance of the individual components, followed by an evaluation of the end-to-end pipelines. The analysis focuses on the quantitative trade-off between accuracy and latency, which is supplemented by qualitative examples.

### 4.1 Overall performance overview

**FIGURE 2:**
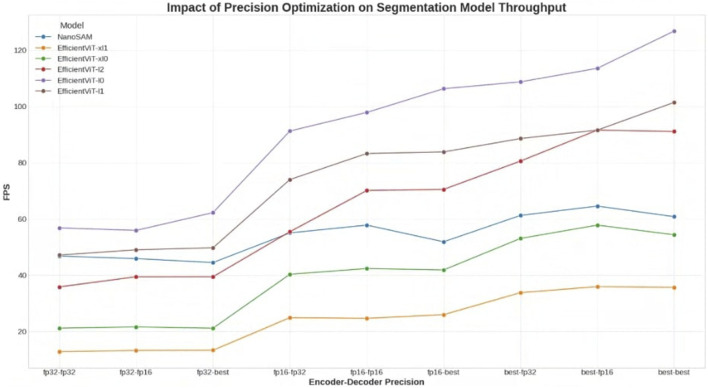
Overall end-to-end performance comparison of the four primary pipeline architectures, measured in Frames Per Second (FPS). Each bar represents the highest throughput achieved for the given combination of an open-vocabulary detector (YOLO-World or NanoOWL) and a prompt-based segmentation model (EfficientViT or NanoSAM). The results clearly indicate that pipelines utilizing the TensorRT-optimized NanoOWL detector significantly outperform those based on YOLO-World, with the NanoOWL + EfficientViT combination achieving the maximum performance.

Figure 2 summarizes the end-to-end performance of the four primary pipeline architectures evaluated in this study. This graph illustrates the optimal performance achievable by each architectural combination.

The most notable result is that pipelines based on the NanoOWL detector exhibit a significant speed advantage over their YOLO-World-based counterparts. Specifically, the highest throughput among all combinations was observed with the pairing of NanoOWL and EfficientViT-SAM. This suggests that the architecture of NanoOWL, fully optimized as a TensorRT engine, operates with high efficiency in a real-world edge device environment.

In contrast, the speed difference between NanoSAM and EfficientViT-SAM in the segmentation stage was found to be relatively minor. Despite employing different lightweight strategies, the impact of these two segmenters on the overall pipeline latency was limited compared to that of the detector.

These findings imply that the end-to-end latency of the pipeline is predominantly determined by the choice of the detection model, while the segmentation model acts as a more influential factor for final segmentation accuracy (mIoU). A detailed quantitative analysis of specific model variants and various precision levels is discussed in-depth in the following Section 4.2.

### 4.2 Component-level performance analysis

The end-to-end pipeline performance differences observed in Section 4.1 originate from the individual characteristics of each component. This section independently evaluates the performance of the two core elements that determine the overall pipeline performance the open-vocabulary object detector and the prompt-based segmentor to analyze the fundamental speed and accuracy characteristics of each architecture.

#### 4.2.1 Open-vocabulary detector performance comparison

The detection model, as the first stage of the overall pipeline, provides the bounding box to the subsequent segmentation module and has a decisive impact on the total latency. This section first conducts an in-depth analysis of the performance of the YOLO-World family, which represents the ‘efficiency-by-design’ approach, and the NanoOWL family, which represents the ‘VLM adaptation’ approach, before presenting a comprehensive comparison of the two architectural philosophies.

First, YOLO-World, executed directly within the PyTorch framework, was evaluated for two of its variants: YOLO-World-S and YOLO-World-X. As presented in Table 1, the YOLO-World-S model recorded an average latency of 26.07 m, while the larger YOLO-World-X model registered 45.59 m. These results indicate that latency is directly influenced by model size and complexity, and underscore the significant performance penalty incurred from the lack of optimization for the edge device.

**TABLE 1:** Comparative analysis of inference latency for open-vocabulary object detectors. The table presents the latency in milliseconds (ms) for two distinct architectural families: YOLO-World (run via PyTorch) and NanoOWL (optimized with TensorRT). Performance for YOLO-World variants is provided as a baseline. For NanoOWL variants, latency is detailed across three different TensorRT precision configurations: FP32, FP16, and the mixed-precision ‘best’ mode.

Model	Latency (ms)		
YOLO-world-S	26.07		
YOLO-world-X	45.59		
NanoOWL (Patch32)	21.23	9.81	10.01
NanoOWL (Patch16)	147.28	39.15	37.13
NanoOWL (Patch14)	1136.36	195.69	198.81

Next, NanoOWL, fully optimized as a TensorRT engine, was evaluated using three models with different backbones across three precision levels: FP32, FP16, and best. For each model, the results are presented in the sequential order of FP32, FP16, and best. Table 1 details the performance of each NanoOWL variant by precision. The fastest model, patch32, achieved a minimum latency of 9.81 m, whereas the heaviest patch14 model was measured at 195.69 m. Notably, a substantial reduction in latency was observed when optimizing from FP32 to FP16. For some models, the ‘best’ mode was observed to yield the optimal speed. This demonstrates that the NanoOWL architecture exhibits high compatibility with TensorRT’s low-precision optimization capabilities.

A comprehensive comparative analysis of the two model families confirms that the NanoOWL series holds a dominant advantage in terms of detection speed. The fastest configuration, OWLViT-base-patch32 at FP16, recorded 9.81 m, showing approximately 2.65 times faster performance than the fastest YOLO-World-S model at 26.03 m. This leads to the critical conclusion that, in our experimental environment, converting models into TensorRT engines to fully leverage the hardware accelerators of the edge device has a decisive impact on performance enhancement.

#### 4.2.2 Prompt-based segmentation model performance comparison

Since the segmentation model receives the bounding box provided by the detector and generates the final segmentation mask, both latency and segmentation accuracy (mIoU) are critical performance indicators. This section provides a multifaceted comparative analysis of the performance of the knowledge distillation-based NanoSAM and the efficient architecture-based EfficientViT-SAM series models.

**FIGURE 3:**
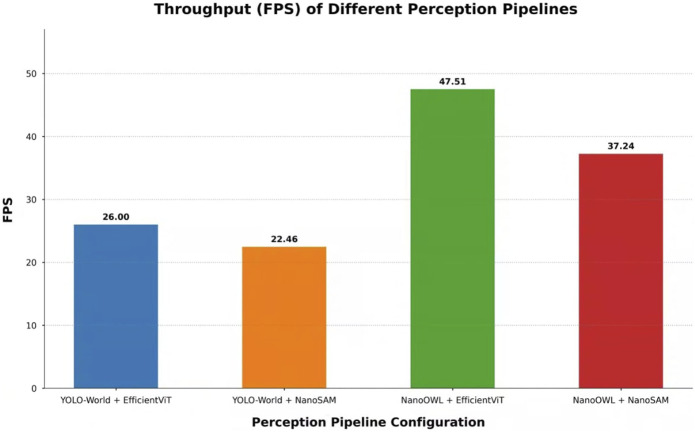
Performance analysis of prompt-based segmentation models across various precision configurations. The chart plots the throughput (FPS) of six segmentation models (five variants of EfficientViT-SAM and NanoSAM) as a function of the precision settings for their respective encoder and decoder components. Precision configurations range from 32-bit floating-point (fp32) to 16-bit floating-point (fp16) and a mixed-precision ‘best’ mode optimized by TensorRT. The results demonstrate a clear trend of performance improvement as precision is lowered, particularly for the encoder.

Figure 3 shows the average latency for the precision of the encoder and decoder of each segmentation model. Although NanoSAM is a single model type, EfficientViT-SAM has a total of five segmentation models, so the graph shows the results for a total of six models. First, in terms of inference speed, Figure 3 shows that the encoder precision of each model improves significantly when going from fp32 to fp16, and from fp16 to best. Overall, it can be confirmed that using the best mode is the fastest. The fastest segmentation model, EfficientViT-SAM-L0, showed a maximum of 17.58 m and a minimum of 7.88 m, while the slowest segmentation model, EfficientViT-SAM-XL1, showed a maximum of 77.8 m and a minimum of 27.78 m. It was also noted that NanoSAM is positioned in the middle.

**FIGURE 4:**
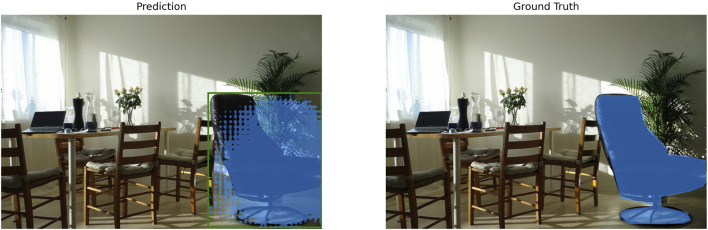
Qualitative example of a successful segmentation result. This figure illustrates a high-accuracy prediction from one of the best-performing pipeline configurations, achieving an mIoU score above 0.8. The generated mask (Prediction) aligns almost perfectly with the ground-truth mask, demonstrating the model’s ability to precisely delineate the target object.

Next, the segmentation accuracy (mIoU) was analyzed not only quantitatively but also in conjunction with the qualitative aspects of the actual segmentation results. As shown in Table 2 a majority of the high-performance configurations, including various precision combinations of the EfficientViT-SAM series, consistently achieved a high mIoU of over 0.8. A representative example of these successful segmentation cases is shown in Figure 4, where it can be confirmed that the object is captured and segmented with great precision.

**TABLE 2:** Performance metrics of EfficientViT-SAM variants under precision settings (FP32, FP16) that yield high segmentation accuracy. The table details the latency (ms) and corresponding high mIoU scores associated with the successful segmentation outcomes illustrated in Figure 3.

Model	Encoder precision	Latency (ms)	mIoU
L0	Fp32	17.16	0.9119
	Fp16	10.19	0.8465
L1	Fp32	20.54	0.9160
	Fp16	12.47	0.9143
L2	Fp32	26.16	0.9167
XL0	Fp32	46.79	0.8846
XL1	Fp32	75.93	0.9186

**FIGURE 5:**
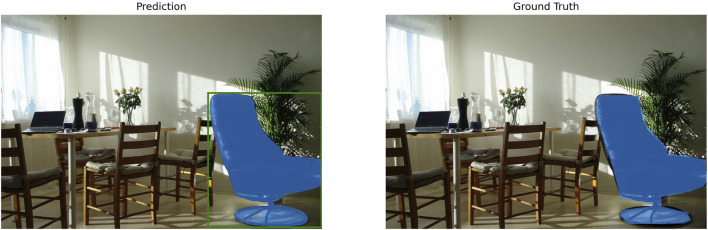
Qualitative example of a partial segmentation failure. This case demonstrates accuracy degradation under aggressive optimization, specifically observed with some EfficientViT-SAM variants using an FP16 encoder, resulting in an mIoU score between 0.4 and 0.5. While the object’s presence is recognized, the predicted mask is imprecise, failing to capture detailed boundaries and including parts of the background.

However, a degradation in accuracy was observed in certain model and precision combinations, which can be broadly classified into two types of failure cases. The first type is the partial segmentation failure case; for the EfficientViT-SAM series models, when the encoder is fp16, all are measured with an mIoU of 0.4–0.5, as can be seen in Figure 5. As shown in the figure, the presence of the detected object was recognized, but the segmentation mask failed to capture the fine-grained boundaries of the object, appearing in a form that included parts of the background or omitted key parts of the object. This suggests that aggressive optimization of the model’s encoder can degrade the model’s generalization performance.

**FIGURE 6:**
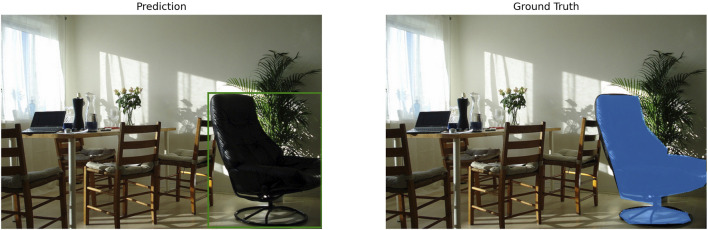
Qualitative example of a complete segmentation failure. This figure illustrates a catastrophic failure case observed with an EfficientViT-SAM model when the encoder was optimized using FP16 precision. The system fails to generate any meaningful segmentation mask for the target object (Prediction), resulting in an mIoU score near zero when compared to the ground truth. This highlights the vulnerability of certain efficient architectures to aggressive quantization strategies.

The second type observed was the case of complete segmentation failure. This phenomenon occurred when the encoders of the EfficientViT-SAM models L0, XL0, and XL1 were optimized with FP16 precision. Figure 5 shows a representative example of this case, where the mIoU score drops below 0.1, resulting in a failure to generate any meaningful segmentation mask. The corresponding quantitative data is presented in Table 3, which confirms a complete failure with an mIoU score of 0 for the L2, XL0, and XL1 variants of EfficientViT-SAM when their encoders are optimized with FP16 precision. This phenomenon underscores the potential brittleness of highly efficient architectures when subjected to aggressive quantization, leading to a total collapse in performance rather than graceful degradation. A qualitative example of this catastrophic failure is illustrated in Figure 6, where the model fails to produce any output for the target object. This visual evidence directly corresponds to the complete failure cases detailed quantitatively in the subsequent text. In contrast, the knowledge distillation-based NanoSAM maintained stable segmentation performance across all evaluated precision optimizations (FP32, FP16, and best), with not a single case of complete failure observed. This demonstrates that NanoSAM possesses high robustness and reliability against aggressive optimization.

**TABLE 3:** Performance metrics of specific EfficientViT-SAM variants where optimizing the encoder to FP16 precision resulted in catastrophic failure. The reported mIoU of 0 for all listed configurations corresponds to the complete failure to generate a meaningful segmentation mask, as illustrated in Figure 5.

Model	Encoder precision	Latency (ms)	mIoU
L2	Fp16	15.48	0
XL0	Fp16	24.06	0
XL1	Fp16	39.66	0

This degradation is quantitatively detailed in Table 4, which shows that while the ‘best’ precision optimization mode yields the fastest inference speeds for the EfficientViT-SAM series, it consistently results in a significant drop in segmentation accuracy, with mIoU scores clustering between 0.42 and 0.54. This highlights a critical trade-off where the pursuit of minimal latency through aggressive mixed-precision optimization can compromise the model's ability to produce precise segmentation masks.

**TABLE 4:** Performance metrics of EfficientViT-SAM variants when applying the ‘best’ mixed-precision optimization to the encoder. This configuration resulted in degraded accuracy, with the reported mIoU scores corresponding to the partial segmentation failure cases illustrated in Figure 4.

Model	Encoder precision	Latency (ms)	mIoU
L0	Best	8.62	0.5108
L1	Best	10.68	0.5238
L2	Best	11.43	0.5306
XL0	Best	18.16	0.4269
XL1	Best	28.44	0.5487

The pronounced difference in robustness between the two segmentation paradigms can be traced back to their fundamental design philosophies. The stability of NanoSAM under aggressive optimization is a direct consequence of its knowledge distillation foundation. In KD, a compact “student” model is trained to emulate the softened output distribution (soft labels) of a larger “teacher” model, not just the ground-truth labels. This process acts as a powerful regularizer, compelling the student model to learn a smoother and more generalized decision boundary. Such a smoothed function is inherently more robust to the discrete perturbations introduced by weight quantization, as minor shifts in parameter values are less likely to cause drastic changes in the output.

Conversely, the brittleness observed in certain EfficientViT-SAM configurations stems from the nature of hardware-aware architectural design. These models achieve efficiency by minimizing parameter redundancy and creating highly specialized computational paths. While exceptionally efficient at full precision, this lack of redundancy means that the information loss incurred during quantization, especially with low-precision formats like FP16, can have a disproportionately large impact. Critical parameters or layers acting as information bottlenecks can be severely degraded, leading to the catastrophic performance collapse observed in our experiments. Therefore, the trade-off is not merely between two models, but between the intrinsic robustness conferred by knowledge transfer and the potential fragility of a highly optimized, non-redundant architecture.

In conclusion, the analysis of the segmentation stage reveals a clear trade-off between the two architectures. The EfficientViT-SAM family, with its variety of models, demonstrated the potential to achieve the highest speeds with certain models and precisions, while simultaneously revealing a vulnerability to unpredictable failures in specific optimization combinations. On the other hand, NanoSAM, although somewhat slower in absolute terms, proved highly reliable by providing consistent performance in terms of accuracy under all optimization conditions. These individual performance characteristics of each component are the direct cause for the overall performance of the end-to-end pipelines, which will be discussed in Section 4.3.

### 4.3 End-to-end pipeline performance analysis

Building upon the preceding component-level analysis, this section evaluates the comprehensive performance of the end-to-end pipelines constructed from the four main architectural combinations. The analysis reveals that the NanoOWL + EfficientViT-SAM combination formed the most superior performance group in all aspects of speed and accuracy. Specifically, the pipeline combining OWLViT-base-patch32 at fp16 with the EfficientViT-SAM-L0 encoder at fp16 and its decoder in best mode achieved an outstanding throughput of 47.51 FPS while maintaining a high mIoU of 84.64%, presenting the most attractive balance between real-time capability and accuracy. In contrast, the YOLO-World-based pipelines recorded a lower FPS overall compared to the NanoOWL-based pipelines. The combination of YOLO-World-S and EfficientViT-SAM-L0, which achieved the fastest throughput among the YOLO-World-based pipelines, recorded 26.68 FPS, a figure that is approximately 43% slower than the fastest NanoOWL-based combination.

A noteworthy point is the impact of the segmentor choice on the final accuracy. Pipelines using NanoSAM, while providing relatively stable mIoU across various optimization conditions, did not reach the maximum accuracy levels of those using EfficientViT-SAM. This is a direct reflection of the trade-offs identified in Section 4.2.2: EfficientViT-SAM possesses higher accuracy potential but exhibits vulnerabilities under specific optimization conditions, whereas NanoSAM has lower absolute accuracy but higher reliability. These characteristics are directly mirrored in the end-to-end performance results.

## 5 Discussion

This chapter synthesizes the experimental results presented previously to discuss their in-depth implications for designing real-time, open-vocabulary perception systems on resource-constrained edge devices. It analyzes the relative merits of competing architectural paradigms and proposes a practical framework for selecting the optimal pipeline according to the requirements of specific HRI scenarios. Finally, it concludes by summarizing the contributions of this work, acknowledging its limitations, and suggesting directions for future research.

### 5.1 In-depth analysis of architectural paradigms

The experimental results of this study clearly demonstrate that the choice of an open-vocabulary detector for edge devices involves a fundamental trade-off that extends beyond static architectural efficiency to the realms of platform-specific optimization potential and linguistic expressiveness.

Despite the complexity of its ViT-based architecture, NanoOWL shows a decisive strength in its ability to be fully converted and optimized into a TensorRT engine. This allows it to leverage the hardware acceleration capabilities of the NVIDIA Jetson platform to their fullest extent, granting it the potential to achieve the lowest latency among the models evaluated in this study. In other words, NanoOWL holds an advantage in terms of optimization potential, making it the definitive choice for applications seeking to secure immediate real-time performance through a proven pipeline. However, this high speed is achieved through an architecture and optimization pipeline tailored specifically for grounding simple noun phrases. The underlying OWL-ViT model was not inherently designed for parsing complex relational sentences, and the NanoOWL framework further specializes the model for this high-throughput, simplified recognition task. This inherent architectural focus is the primary reason for its structural limitations in understanding complex states a deliberate trade-off to achieve state-of-the-art latency on edge hardware, representing the pinnacle of hardware optimization.

In contrast, YOLO-World, in the PyTorch file format used in this study, falls short of NanoOWL in terms of speed but possesses a dominant advantage in linguistic expressiveness. This model is designed to understand complex, sentence-level referring expressions, enabling it to “ground” relationships between objects, such as in “the person closest to the door,” to the visual world. This is a critical capability that can elevate human-robot interaction beyond simple object designation to a much more natural and sophisticated level of communication. Therefore, YOLO-World demonstrates its value in high-level HRI scenarios where sophisticated language understanding is more critical than immediate speed. However, this model is not designed to be compatible with TensorRT optimization; attempting to do so would result in the loss of its zero-shot capabilities.

In conclusion, the two detector paradigms are optimized along different axes. NanoOWL represents the pinnacle of ‘hardware optimization,’ while YOLO-World represents the pinnacle of ‘language capability optimization.’ Robotics system designers must clearly recognize this trade-off and make a strategic choice based on the core capabilities required by their application.

### 5.2 A framework for HRI scenario-based pipeline selection

Based on this new analysis, the guidelines for pipeline selection according to the requirements of HRI applications can be redefined as follows.

For High-Responsiveness Tasks, such as object tracking or dynamic obstacle avoidance where latency is the most critical factor, a NanoOWL-based pipeline is undoubtedly the most suitable choice. Fully accelerated by its TensorRT engine, NanoOWL provides the highest FPS and delivers optimal performance in environments that require rapid recognition based on clear noun phrases like “a car” or “a person.”

For High-Level Language Understanding Tasks, such as precise manipulation scenarios that require understanding complex context or relationships like “pick up the cup I am looking at,” a YOLO-World-based pipeline is the only viable option. Although it may be slower due to its PyTorch framework basis, semantic accuracy takes precedence over speed, as the task itself would fail if the command is not understood.

A Balanced Sweet Spot still exists for general HRI tasks that require an adequate level of speed (>15 FPS), high mIoU, and language capabilities beyond basic noun phrases. The pipeline combination and precision level that best satisfy this balance should be selected from the Pareto front of the experimental results.

### 5.3 Limitations and future work

The limitations of this study and corresponding directions for future research are as follows. First, a primary limitation of this study is its evaluation on a static image dataset, the RefCOCO+. This approach does not fully account for the temporal complexities and dynamic challenges, such as motion blur or varying lighting conditions, that arise in a real robot’s video stream. Future work should therefore extend this analysis by deploying the most promising pipelines on a physical mobile manipulator. This would enable a comprehensive evaluation of their real-world robustness and performance within a complete “command-to-action” loop, as planned in our future research trajectory. Second, the focus was exclusively on the perception module, and integration with downstream decision-making and control stacks was not addressed.

A key future research direction is to extend the OWL-ViT model, the backbone of NanoOWL, to enable the understanding of full sentences in addition to noun phrases, while still maintaining full optimization through TensorRT. If a model based on OWL-ViT could recognize sentences at its current speed, it has the potential to become a dominant solution possessing both linguistic expressiveness and speed. This would be a significant milestone for next-generation perception pipelines in edge robotics. Furthermore, this work can be extended to deploying the identified optimal pipeline on a physical mobile manipulator to evaluate the performance of the full “command-to-action” loop, and to research aimed at achieving Vision-Language-Action (VLA) capabilities for processing and executing more complex commands on edge devices (Brohan et al., 2023; Lee et al., 2024).

### 5.4 Conclusion

This study presented a comprehensive benchmark of open-vocabulary perception pipelines on the NVIDIA Jetson AGX Orin and identified a critical trade-off between hardware optimization potential and linguistic expressiveness. The TensorRT-accelerated NanoOWL provided the fastest performance, though limited to noun-phrase recognition, while the PyTorch-based YOLO-World was relatively slower but demonstrated superior language capabilities by understanding complex sentences. Through this quantitative and qualitative analysis, this research provides empirically-grounded guidelines that enable robotics researchers and engineers to make informed decisions in the selection and development of architectures tailored to their specific HRI requirements.

## Data Availability

The original contributions presented in the study are included in the article/supplementary material, further inquiries can be directed to the corresponding author.
